# Assessing Intestinal Health in Pigs: Recognizing Unaddressed Areas and Prospective Research Avenues

**DOI:** 10.3390/vetsci12050475

**Published:** 2025-05-14

**Authors:** Marina Patricia Walter, Gabriela Miotto Galli, Alicia Zem Fraga, Aires Santos Silva, Júlio César Vieira Furtado, Pedro João Viera Ascari, Ines Andretta

**Affiliations:** 1 Department of Animal Science, Universidade Federal do Rio Grande do Sul, Porto Alegre 91540-000, Rio Grande do Sul, Brazil; 00273634@ufrgs.br (M.P.W.); gabi-gmg@hotmail.com (G.M.G.); 00340711@ufrgs.br (A.S.S.); julio.furtado@ufrgs.br (J.C.V.F.); pedro.ascari@ufrgs.br (P.J.V.A.); 2 Department of Animal Science, Universidade Federal Rural do Rio de Janeiro, Seropédica 23890-000, Rio de Janeiro, Brazil; azfraga@ufrrj.br

**Keywords:** finishing, intestinal integrity, invasive methods, non-invasive methods, nursery, semi-invasive methods, swine

## Abstract

Gut health is crucial for pigs because it affects nutrient absorption, immune function, and growth performance. However, its definition remains unclear, particularly regarding measurable responses in scientific studies. This review critically analyzed studies assessing intestinal health in pigs during the nursery, growing, and finishing phases, focusing on the invasiveness of the applied techniques. Papers published between 2005 and 2023 were searched in PubMed, Web of Science, and Science Direct, resulting in 322 articles. Publications grew at an annual rate of 23%, with 84% of studies conducted in the nursery phase. In the database, 87% of intestinal health assessments were combined with growth performance measures. Semi-invasive methods, such as blood collection, were used in 65% of the studies. Non-invasive measures, such as fecal samples, were analyzed in 39% of the studies, whereas urine (7%) and saliva (0.6%) were used in only a small fraction of the studies. Invasive methods, including intestinal tissue collection, were employed in 89% of studies. Among these, 24% did not specify the euthanasia method or number of pigs euthanized. No significant association was found between invasive and non-invasive methods, limiting the ability to establish clear relationships. Future studies should integrate multiple levels of invasiveness to improve the understanding and reduce reliance on invasive techniques.

## Simple Summary

Gut health is vital for pigs, as it influences nutrient absorption, immunity, and growth. However, defining and measuring it remains a challenge. This study reviewed 322 scientific papers to evaluate the assessment of intestinal health, focusing on the invasiveness of different methods. Most studies have relied on invasive techniques, such as tissue collection, while non-invasive approaches, such as feces, urine, and saliva analysis, are less common. No clear link was found between invasive and non-invasive methods, highlighting the gaps in the current studies. Future studies should combine different approaches to improve gut health assessment while reducing the need for invasive procedures. This could enhance animal welfare and lead to better pig production strategies.

## 1. Introduction

Intestinal health is an important and recent topic in swine research. Functional and healthy gastrointestinal tracts are critical for pig welfare and production efficiency at every stage of life [1]. The term intestinal health refers to the set of main components associated with gastrointestinal functionality. This definition combines the main points of intestinal health, that is, it evaluates factors associated with diet, structure, and function of the gastrointestinal tract barrier, normal and stable microbiota, and health status (absence of gastrointestinal disease), in addition to effective digestion of feed and absorption of nutrients [2,3].

Gut health can be significantly affected by several factors, including environment, feed quality, and animal management. Diet composition (e.g., ingredients, nutrients, and additives) and quality (e.g., mycotoxins) can also influence the development and function of the digestive system, including the immune system and microbiota [4]. For this reason, it is widely studied in research projects aiming to optimize pig performance.

Concerns about swine gut health are particularly important in the post-weaning period, when piglets face a series of challenges that can compromise intestinal integrity. For instance, low feed consumption indicates a lack of luminal nutrition, which can influence intestinal health [5]. Furthermore, changes in the environment, social groups, and feeding patterns are associated with changes in the structure and function of the intestinal tract [6]. Considering these factors, gut health modulation may play a crucial role in reducing the need for antimicrobials and protecting animals from diseases during this challenging phase. However, gut health remains important during the growing–finishing phase. Although this stage is not as demanding as the nursery phase, it accounts for most of the feed consumption in pig production, significantly impacting the economic performance.

Despite being widely studied, gut health remains a multifaceted concept assessed through different sets of variables across various research contexts. In view of the high number of articles published on intestinal health in pigs in the last decade, a comprehensive analysis is essential to unravel the complexity of this field. Therefore, this review critically explores the assessment of gut health in pigs through various studies, analyzing evolving trends in the scientific literature, with a particular emphasis on the invasiveness of the applied techniques.

## 2. Materials and Methods

This study was conducted in accordance with PRISMA guidelines for systematic reviews, with all criteria applied during the search and selection methodology. During the critical analysis of the results, the approach was adapted to a narrative approach, while maintaining impartiality and completeness in the evaluation process.

Digital databases (PubMed, Web of Science, and Science Direct) were searched to identify studies published in scientific journals that reported the methods used to investigate intestinal health in pigs. The keyword ”gut health” combined with ”pigs” was used in the search. Alternative terms were listed using synonymous words in English to compose the final search. The final search terms were as follows: (pig OR pigs OR swine OR piglet*) AND (“intestinal health” OR “gut health” OR “intestinal-health” OR “gut-health” OR “intestinal function” OR “intestinal integrity” OR “gut function” OR “gut integrity”).

The main criteria for paper selection were as follows: (a) full papers published in scientific journals from 2005; (b) use of the term ”gut health” or ”intestinal health” in the title or abstract; and (c) evaluation of pigs from nursery to finishing rearing phases. The timespan began in 2005 because of the limited number of relevant studies published before that year; only five were identified prior to 2005, whereas publications became more consistent from that point onward. A literature search was conducted in December 2023. The databases were exported to reference management software (EndNote X9, Philadelphia, PA, USA), in which the next steps of selection were performed.

**Figure 1:**
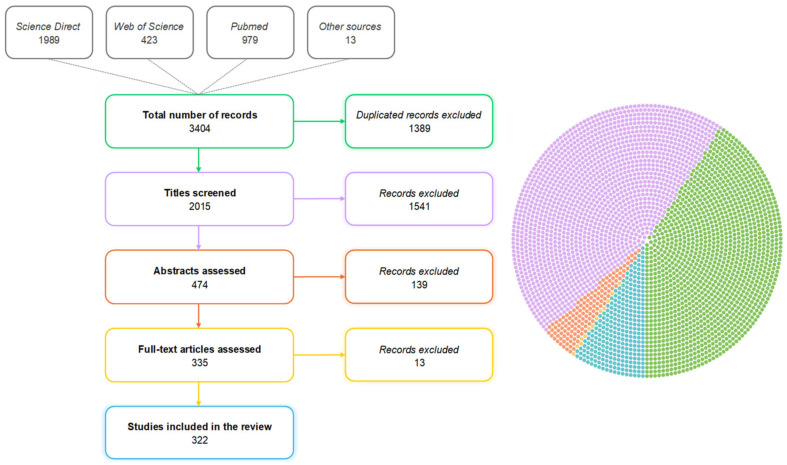
Flow diagram showing the methods used to select studies on intestinal health in pigs. The total pie chart represents the initial number of articles retrieved in the search (n = 3404) and the fractions corresponding to each step of the flow diagram, using matching colors for easier interpretation. Chart colors: green, the total number of records and duplicates excluded; Purple, titles screened and records excluded; Orange, abstract assessed and records excluded; Yellow, full text article assessed and records excluded; and blue, the final studies included in the review.

The initial database comprised 3404 references (Figure 1), of which duplicate references (1389) were excluded. The titles and abstracts were assessed, resulting in the exclusion of 1541 and 139 references, respectively. Titles were excluded if they clearly referred to non-experimental studies, non-swine species, or topics unrelated to intestinal health. Abstracts were excluded if they were not related to intestinal health or if they referred to review articles or in vitro studies. Full texts were excluded when the methods or outcomes were not clearly described, when the study was not conducted in pigs, or when the article was not available in English. Later, 335 papers were fully evaluated individually by three members of the team. Any additional removal was discussed with the team and, if accepted, was registered in the PRISMA flow diagram. After this last phase, 322 publications were selected, and all relevant information related to the proposed theoretical model was transferred from these documents to an electronic spreadsheet.

A list of all responses evaluated for each study was created. Methods using tissues (e.g., morphology, gene expression, cellular presence, and oxidative stress) were classified as invasive, whereas those using blood (e.g., oxidative stress, acute-phase proteins, gene expression, biochemical parameters, and hemogram) were classified as semi-invasive. Finally, research techniques based on fecal and urinary samples were classified as non-invasive.

Growth performance was assessed as body weight, average daily gain, feed intake, feed-to-gain ratio, and gain-to-feed ratio. Urinary responses included mainly excretion of lactulose, mannitol, and nitrogen, along with participation in nutrient metabolism tests. Fecal analyses were mainly conducted to quantify the presence of microorganisms, the overall microbiota composition, along with participation in nutrient digestibility tests. Intestinal morphology was evaluated by measuring the villus height, villus width, villus area, crypt depth, villus-height-to-crypt-depth ratio, and mucosal thickness. Additionally, goblet cells, lymphocytes, enterocytes, and macrophages were counted. Gene expression in intestinal tissues was analyzed for key inflammatory and structural markers, including IL-1α, IL-1β, IL-6, IL-8, IL-10, IL-17, TNF-α, IFN-γ, TGF-β, Caspase-3, HSP70, NF-κB, MYD88, TLR4, as well as tight junction and barrier-related genes, such as Occludin, Claudin-1, Claudin-2, Claudin-3, ZO-1, ZO-2, mucin genes (MUC1, MUC3, and MUC4), along with housekeeping genes, including GAPDH and β-actin (Bactin). Other genes related to nutrient absorption and antioxidant regulation, including SGLT1 and NRF1, were also evaluated. Oxidative stress was assessed by measuring total antioxidant capacity, superoxide dismutase, malondialdehyde, reactive oxygen species, reduced glutathione, glutathione peroxidase, and thiobarbituric acid reactive substances. The acute phase response was monitored via serum levels of C-reactive protein, haptoglobin, lipopolysaccharide-binding protein, serum amyloid A, and total iron-binding capacity. Blood biochemical responses included urea nitrogen, total protein, glucose, creatinine, albumin, cholesterol, triglycerides, alkaline phosphatase, alanine aminotransferase, aspartate aminotransferase, gamma-glutamyl transferase, non-esterified fatty acids, and insulin. Intestinal permeability was evaluated using markers such as D-xylose, mannitol, diamine oxidase, intestinal fatty acid-binding protein, and FITC-dextran. Finally, hematological and immunological assessments included total and differential blood cell counts, including white blood cells, red blood cells, hemoglobin, hematocrit, mean corpuscular hemoglobin, platelets, leukocytes, basophils, monocytes, neutrophils, lymphocytes, eosinophils, and T-cell subpopulations, including CD3+, CD4+, and CD8+ cells. Although the list is quite extensive, it does not encompass all the responses observed in the studies, but rather highlights the most frequently reported ones.

Cross-study comparisons were performed by considering the subject, scope, and main responses assessed in the studies. Graphical, correlational, and descriptive analyses were performed. Pearson’s chi-squared test for association was used to assess the relationship between the types of research techniques applied in the studies (*p* ≤ 0.05). This statistical test evaluated the association between the presence or absence of specific types of responses (e.g., whether a study measured growth performance), rather than analyzing the magnitude of the outcomes themselves (e.g., the amount of weight gain observed). All tests were conducted using Minitab v.18 software (State College, PA, USA).

## 3. Results

**Figure 2:**
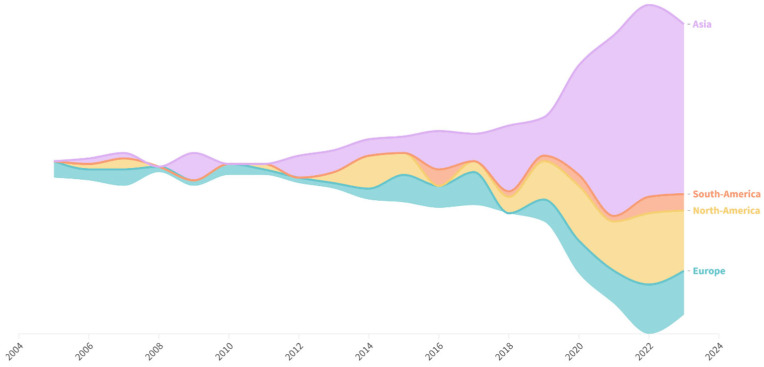
Timespan of publications on pig gut health and regional contributions to the database. As a reference for scale, 3 studies were published in 2005, and 60 studies were published in 2022.

The database comprises 322 scientific papers published between January 2005 and November 2023. The annual evolution of the publications during this period is reported in Figure 2. The average annual growth rate was 22.9%, with an average of seventeen articles published per year. In particular, production increased exponentially from 2014 to 2023, with 2022 being the most prolific year with 60 published articles. Additionally, production increased considerably between 2019 and 2020, from 19 to 38 articles.

Researchers from 35 countries authored these publications. Considering only the country of the corresponding author, the top five countries were China (46.4%), the United States (20.6%), Ireland (5.3%), Spain (4.0%), and Brazil (3.7%). Most studies (75%) involved researchers from a single country, whereas the remaining studies included international collaborations. Among these, researchers from the United States were the most frequent collaborators in papers with primary authors from other countries. At least one researcher from a private company was listed as the author of the 72 articles. The highest percentage of authors affiliated with private companies was found in studies published in China (29.0%) and the United States (31.0%). A single institution was responsible for the project in 32% of the reviewed publications. Two institutions collaborated in 26.0% of the studies, whereas 23.0% involved three institutions. The remaining articles featured collaborations among four or more institutions.

**Figure 3:**
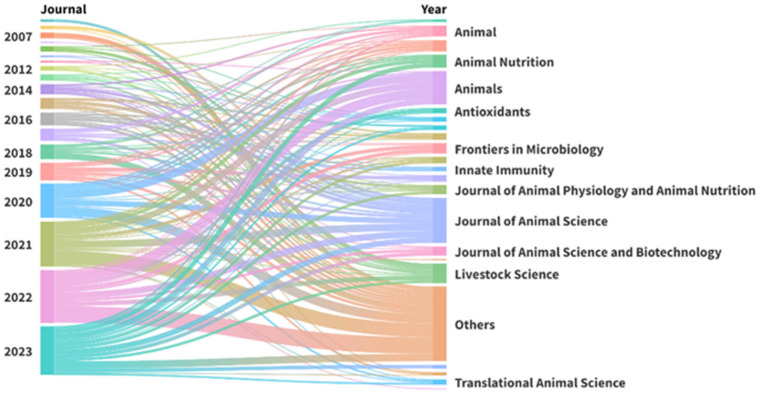
Distribution of publications on pig intestinal health across journals (right) and years (left) from 2007 to 2023. Each color represents a different journal, with “Others” grouping journals with fewer publications. As a reference for scale, 60 studies were published in 2022 (first axis), and 49 studies were published in the *Journal of Animal Science* (second axis).

**Figure 4:**
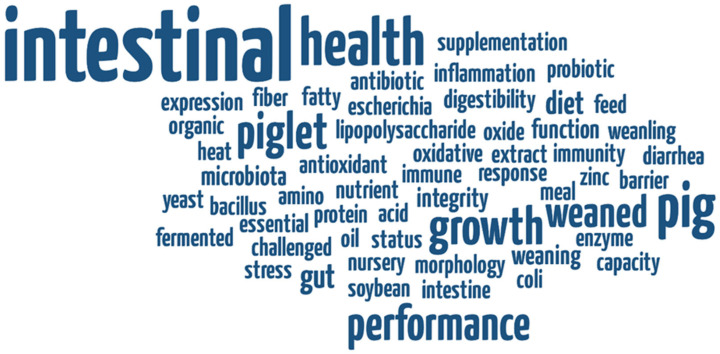
Key research terms used in the database of intestinal health in pigs (word frequency based on title and keywords).

The selected papers were published in 94 scientific journals (Figure 3). The most frequent sources were *Journal of Animal Science* (49 papers), *Animals* (36 papers), *Livestock Science* (21 papers), and *Animal Nutrition* (15 papers). The most frequently used keywords were ”intestinal”, ”performance”, ”growth”, ”health”, ”pig”, and ”weaned” (Figure 4).

Only 16.1% of the studies were conducted during the growing–finishing phase. Furthermore, the vast majority (83.9%) of the studies were conducted during the nursery phase (classification was performed considering the terminology used by the original authors). Barrows and females were used in 65% of the studies; 27% of the trials used only barrows, and 7.5% used only females.

**Figure 5:**
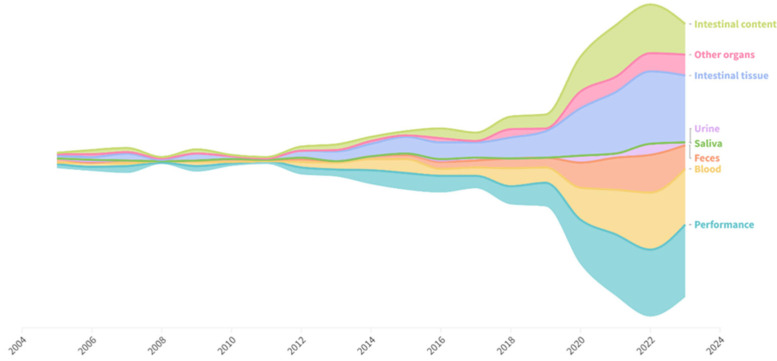
Samples used in the pig intestinal health database.

Performance responses were assessed in 87% of studies (Figure 5). Semi-invasive measures, such as those involving blood collection, were used in 64.9% of studies. Non-invasive measures have become more frequent in the last decade, with a notable increase in the use of fecal samples, which were analyzed in 39.1% of the studies. In contrast, only a small fraction of the studies used urine (7.5%) and saliva (0.6%). Invasive methods were used in most studies. Intestinal tissues, particularly those from the small intestine, were collected and assessed in 88.8% of the studies. Only 22.3% of the studies collected tissues from other organs, with the most frequently collected tissues being from the liver, spleen, and stomach. Among the studies that included invasive methods and required euthanasia in their protocols, 25.7% did not specify the method used and/or the number of pigs euthanized. The most commonly reported method of euthanasia was sodium pentobarbital. Other methods reported included electrical stunning and captive bolt stunning.

In the five leading countries, in terms of publications (frequency), semi-invasive and non-invasive methods (i.e., those involving blood, feces, and urine) were used to assess intestinal health in 82.0% of the studies in China, 72.0% in the United States, 29.0% in Ireland, 62.0% in Spain, and 75.0% in Brazil. Invasive methods were more frequently employed, appearing in 91.0% of studies in China, 83.0% in the United States, 59.0% in Ireland, and 92.0% in Spain and Brazil.

**Figure 6:**
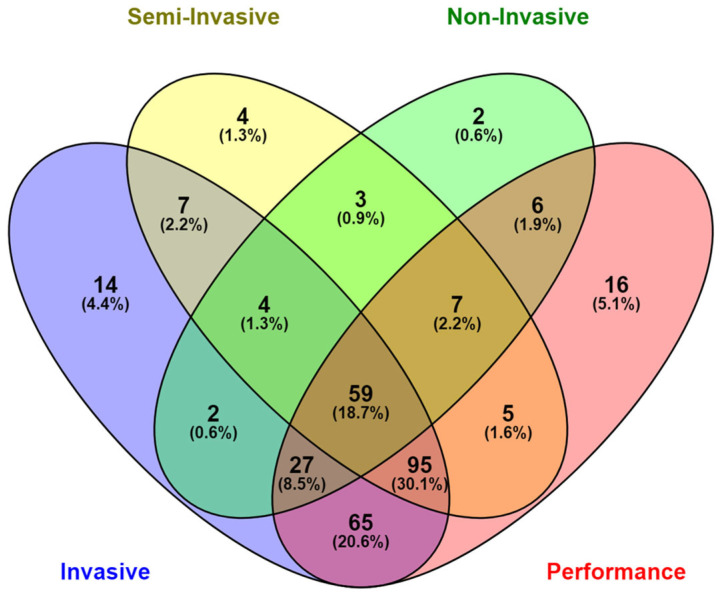
Association among the degree of invasiveness of the responses in studies that compose the database of intestinal health in pigs. Colors represent different levels of invasiveness: purple for invasive, yellow for semi-invasive, green for non-invasive, and red for growth performance variables.

Integrating multiple techniques within a single study may enhance the understanding of gut health by providing a broader and more detailed assessment. In this regard, 59 studies (18.3%) combined growth performance measures with invasive, semi-invasive, and non-invasive responses (Figure 6). Invasive and non-invasive responses were combined in only 0.6% of the studies. Corroborating these descriptive results, a significant association (Pearson’s chi-square test for association) was found between invasive responses and growth performance (*p* < 0.001), as well as between invasive and semi-invasive responses (*p* = 0.007). However, no association was observed between invasive and non-invasive responses (*p* = 0.609), indicating that these types of responses do not commonly appear together in these studies.

**Figure 7:**
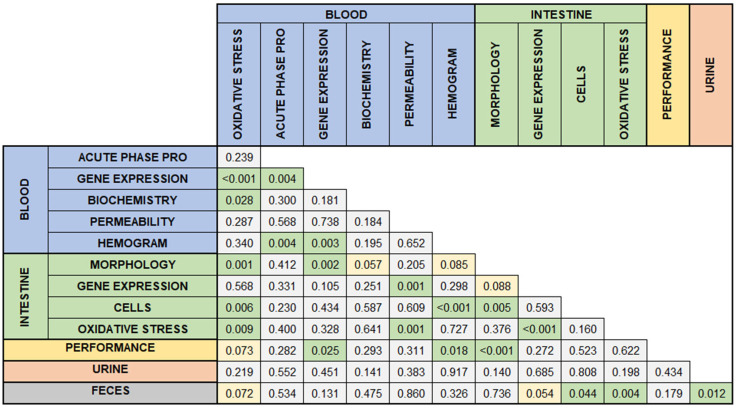
Associations among sample collection sites in studies comprising a database of intestinal health in pigs. The associations were considered statistically significant at *p* < 0.05. Green indicates *p* ≤ 0.05; yellow indicates 0.01 < *p* ≤ 0.05.

Several associations were observed between the presence and absence of specific types of responses (Figure 7). Growth performance was associated with blood gene expression (*p* = 0.025), hemogram (*p* = 0.018), and intestinal morphology (*p* < 0.001). Additionally, oxidative stress in the blood was associated with oxidative stress in the intestine (*p* = 0.009). Urinary assessments were associated only with fecal analyses, which in turn were linked to cellular and oxidative stress evaluations in the intestines.

## 4. Discussion

The availability of publications focusing on the intestinal health of pigs has been extensively explored in the last few decades. The first studies selected for these meta-analyses were published in 2005; however, the availability of studies focusing on pigs evolved significantly in the following years, especially after 2020. This pattern can also be identified at conferences and at scientific events. Research subjects related to intestinal health made up a significant portion of the presentations (35.0%, 102 presentations) at two major international conferences in 2023 [7].

A previous study encompassing 11 animal categories identified pigs as the group with the highest number of publications on intestinal health [8]. Additionally, most articles in this field are related to dietetics and nutrition, underscoring that gut health is an increasingly prominent research topic. This trend is likely driven by the ban on antimicrobial growth promoters in animal feed. In 2006, the European Union (Reg. No. 1831/2003/EC) banned the use of antibiotics as growth promoters in animals. Since then, other countries have developed policies to reduce or ban antibiotic use in animal production, which has directly affected farm management practices [9]. Many antibiotic alternatives, including enzymes, probiotics, prebiotics, inorganic acids, medicinal plants, immunostimulants, and management practices, have been used to enhance animal health and growth performance. Many of these alternatives are directly related to intestinal health, thus justifying the importance of research in this area.

The most prolific journals in the database were *Journal of Animal Science* (15.3%), *Animals* (11.2%), and *Livestock Science* (6.5%). These are all English language journals, and the first two are included in the first quartile for *Animal Science* and *Zoology*, according to the 2024 *Journal Citation Reports* (JCR). *Animals* and *Livestock Science* are also included in the first quartile, but only in the veterinary field. The *Journal of Animal Science* encompasses a broad range of topics related to agricultural and biological sciences, such as biochemistry, genetics, molecular biology, and medicine. *Animals* and *Livestock Science* have focused more on the agricultural, biological, and veterinary sciences. Pigs can also serve as a model for human health research. However, only a limited number of studies were found in journals focused on this field.

Most studies were conducted in Asia, with China accounting for 46.4% of the total. The United States was the second largest contributor, accounting for 20.6% of the studies. This aligns with the fact that China and the USA are the leading countries in pig production [10], which naturally drives greater research efforts in this field. The prevalence of invasive methods in these countries may also reflect differences in national legislation. In the USA, animal experimentation is regulated by the Animal Welfare Act (AWA) and overseen by Institutional Animal Care and Use Committees (IACUC). In China, although ethical oversight has expanded in recent years, regulations such as the “Guidelines for the Humane Treatment of Laboratory Animals” tend to be more flexible regarding livestock species. The countries with the highest numbers of studies were Ireland, Spain, and Brazil. In the specific case of Brazil, pork production has increased over the last few decades, becoming one of the main pork producers in the world [11].

As expected, the most frequently used keyword was intestinal health. Animal growth performance is the primary parameter used to evaluate intestinal health. Other areas of interest include dietary supplements and oxidative stress. The key concepts highlighted in this study were oxidative stress, microbiota, intestinal integrity/morphology, nutrient digestibility, and diarrhea. All of these terms are interconnected. The gastrointestinal tract, together with the liver, is important for the growth, development, and health of farm animals. In the first pass, these organs determine the post-absorptive metabolism of nutrients [12]. Recent studies emphasize that the composition of the gut microbiota or microbiome plays a crucial role in maintaining intestinal health. It influences nutrient absorption, feed digestibility, energy harvest, and consequently animal productivity [6,13,14].

Most studies were conducted in the nursery phase, while only 16.1% developed in the finishing phase. The nursery is one of the most challenging phases for weaned pigs, as they are subjected to environmental and physiological changes such as abrupt separation from the sow, difficulties in adapting to the pen, and mixing with piglets from other litters. The determination of a new hierarchy, as well as the sudden change in diet, are factors considered stressful, which also determine important challenges for subsequent survival [15]. The digestive system is considered the largest organ of the immune system because it houses more than 70.0% of the cells of the immune system [16]. It is worth mentioning that gut development in weaned pigs is sensitive to alterations in feed components, as reflected by their morphological changes [17]. Because of these factors, studying changes in the nursery phase is essential, but more studies in the finishing phase are necessary to understand how intestinal challenges affect long-term gut health and growth performance.

Growth performance is closely associated with several physiological and intestinal parameters such as oxidative stress, gene expression, hematological profile, and intestinal morphology [18]. These indicators provide a deeper understanding of the underlying mechanisms that influence animal health and productivity efficiency. Morphological analyses, including villus height, crypt depth, and villus-to-crypt ratio, are considered the gold standards for evaluating gut structure and function [19]. Additionally, the evaluation of gene expression and oxidative stress markers offers insights into cellular responses to nutritional or environmental challenges. While these methods require invasive sampling, they were employed in 88.8% of the reviewed studies, highlighting their relevance despite logistical and ethical limitations in field conditions.

Moreover, the presence of specific cell types (e.g., goblet cells and immune cells) and changes in the intestinal microbiota have been increasingly studied owing to their critical role in modulating nutrient absorption, immune response, and gut barrier integrity. These components are strongly interconnected with growth performance, as a balanced gut microbiome and regulated immune environment can directly affect nutrient utilization and health outcomes. However, evaluating such parameters in commercial settings remains challenging because of the complexity and cost of the required methodologies.

Feces, urine, and saliva are considered non-invasive methods. Urine was rarely used; however, many studies have collected feces, mainly to analyze digestibility. The evaluation of digestibility through fecal analysis enables the measurement of nutrient absorption efficiency and the identification of potential gastrointestinal dysfunctions. However, feces represent the end-product of the digestive process and thus contain much more information on both the feed itself, the history of its transit through the animal digestive tract, and the health status of the pig [20]. The frequency of fecal sample collection is high, but its analytical potential remains underutilized. Various intestinal health biomarkers can be assessed through this non-invasive method but are still rarely explored. The same applies to blood samples collected in most studies; however, analyses related to intestinal permeability and blood biomarkers remain limited. This approach is non-invasive and easy to implement in production systems, allowing for early identification of metabolic and nutritional disorders, so it is a sample that can be widely used to evaluate gut health.

Associations were identified among the variables. When the association was significant, these responses appeared together. In our analysis, we found a significant association between invasive and semi-invasive methods. However, no significant association was observed between the invasive and non-invasive methods. This means that when reviewing these studies, it is not possible to find sufficient information in the literature to analyze the relationship between these variables. This is because studies evaluating intestinal health through invasive methods did not assess non-invasive variables.

Nowadays, the welfare of animals, especially those intended for food production, is gaining increasing relevance. Societal views on scientific experiments conducted are predominantly negative. Consequently, invasive experimental procedures can be restricted or prohibited. Therefore, experiments should be conducted with higher ethical standards and lower invasiveness [21].

To reduce animal euthanasia in experiments, it is essential to understand whether intestinal health responses assessed using blood, feces, and urine are truly comparable to those evaluated using intestinal tissue. However, it is still a challenge to find these answers, as studies often fail to provide this information collectively. It is crucial for future research to evaluate non-invasive responses to include invasive variables for a transitional period. This approach would allow the creation of a comprehensive database for analysis, paving the way for establishing a reliable association between methods in the future.

Intestinal health is associated with a set of factors, and several analyses are necessary to define it. It is important to highlight that several studies have mentioned some important factors (e.g., methods of collection and microbiota) in their methodologies as important characteristics to be considered in determining intestinal health, as well as in animal growth performance in pig production. Many studies have shown that gut health is a relevant research area in animal production and veterinary medicine, with a constant increase in publications, especially over the last ten years. This allowed the selection of criteria for the formation and homogenization of the database and a better understanding of the term intestinal/gut health.

## 5. Conclusions

The growing interest in pig gut health underscores the importance of enhancing nutrition and production efficiency. Although most research has focused on the nursery phase, further investigation is needed during the growing-finishing phase to better understand its effects on gut health. The key parameters for assessing intestinal health include intestinal morphology, gene expression, oxidative stress, and microbiota composition. Semi-invasive and non-invasive sampling methods, such as blood, feces, urine, and saliva, represent promising tools for gut health assessment, yet they remain underexplored. Future research should prioritize the integration of these techniques, not only to enhance animal welfare but also to strengthen the accuracy and applicability of gut health indicators in modern swine production.

## Data Availability

No new data were created.
